# Real-world efficacy of long-term teduglutide use in pediatric patients with short bowel syndrome

**DOI:** 10.1016/j.intf.2025.100312

**Published:** 2025-10-23

**Authors:** Claire Josey, George Mazariegos, Elizabeth King, Pamela Holzer, Jeffrey Rudolph, Vikram Kalathur Raghu

**Affiliations:** a Department of Pediatrics, University of Pittsburgh Medical Center (UPMC) Children’s Hospital, Pittsburgh, PA, USA; b Department of Surgery, Hillman Center for Pediatric Transplantation and Thomas E. Starzl Transplantation Institute, UPMC Children's Hospital of Pittsburgh, Pittsburgh, PA, USA

**Keywords:** Short bowel syndrome, Teduglutide, Intestinal failure, Parenteral support

## Abstract

**Background:**

In patients with short bowel syndrome (SBS), teduglutide reduces dependency on parenteral support (PS) by promoting intestinal growth and absorption. We aim to describe long-term outcomes using teduglutide in a large pediatric intestinal rehabilitation and transplant center.

**Material and Methods:**

We performed a single-center, retrospective analysis of teduglutide use in patients with SBS ages 1–23 years. Sex, age, intestinal length, BMI, PS regimens, and isolated small bowel (ISB) transplant status are described. Subgroup analysis comparing younger and older patient cohorts was performed. Primary end point was a reduction in PS volume of ≥ 20 %.

**Results:**

27 patients (10 female; mean age 8.9 years) received subcutaneous teduglutide (0.05 mg/kg/d) once daily for mean 120.8 weeks. Mean bowel length was 56.9 (SD=52.1) cm. 5 were listed for ISB transplant, 2 were “inactive,” and 20 were not listed. 2 patients were post-enterectomy.

A decrease in PS volume of ≥ 20 % was experienced by 67 % of patients, with 30 % reaching enteral autonomy at mean 71.57 (SD=56.37) weeks (Fig. 1). Significant decreases in PS volume, hours and days, nonprotein calories, parenteral nutrition dependency index (PNDI), and lipid dose were observed (Table 2). Of patients listed at baseline, 2 remained listed, 2 were removed, and 1 was reclassified as “inactive.” 1 was newly listed and 2 “inactive” patients were removed. 19 remained unlisted.

The most frequent adverse event was pyrexia. 10 patients permanently discontinued treatment at mean 62.59 (SD=55.55) weeks, 4 due to related AEs.

**Conclusion:**

Pediatric SBS patients experienced significant decreases in PS dependency, providing real-world evidence of teduglutide efficacy.

## Introduction

Short bowel syndrome (SBS) results from surgical resection or congenital defects and the subsequent loss of absorption, frequently necessitating dependency on parenteral support. Treatments which increase intestinal absorption present the opportunity to minimize dependency on parenteral support.

Glucagon-like peptide-2 (GLP-2) is a naturally-occurring hormone secreted by intestinal cells during digestion. GLP-2 promotes epithelial proliferation and intestinal surface area. [1], [2] Administration of teduglutide, a GLP-2 analog, enhances villous height and crypt depth and aims to reduce dependency on parenteral nutrition. [2] Teduglutide is approved for the treatment of pediatric patients with SBS with parenteral support dependency in North America, Europe, and Japan. [3] A pooled analysis of two open-label phase 3 studies investigating the efficacy and safety of teduglutide in 78 children with SBS found that 82 % of patients achieved a significant clinical response, defined as a 20 % reduction in parenteral nutrition or intravenous volume. 22 % of these patients achieved enteral autonomy at 96 weeks.

This report describes the real-world experiences of pediatric patients receiving long-term teduglutide treatments. This large pediatric intestinal rehabilitation and transplant center did not participate in clinical trials for teduglutide. Additionally, this study aims to identify factors that are predictive of treatment success or failure, which will contribute to clinical decision-making regarding the initiation and withdrawal of teduglutide treatment.

## Material and Methods

We performed a retrospective, cross-sectional study of teduglutide use in children with SBS at a single center. The center’s intestinal rehabilitation team includes three providers who make independent clinical decisions and share patient caseloads. There is no formal protocol utilized for weaning PS; decisions including initiating and discontinuing teduglutide treatment are discussed in weekly conferences consisting of physicians, surgeons, nurses, and social workers.

### Patient population

Inclusion criteria included a) past or current patients, b) diagnosis of SBS, c) documented dependency on parenteral support, including total parenteral nutrition (TPN) and/or IV fluids, and d) daily injections of teduglutide for at least three weeks.

Patients were excluded on the basis of noncompliance, use of teduglutide for less than three weeks, or a lack of documented dependency on parenteral support.

A retrospective chart review was conducted on the final cohort. Eligibility for teduglutide was determined through a safety assessment which included a colonoscopy.

### Data collection

Demographic and clinical data were obtained from the electronic medical record system. Baseline characteristics included age, sex, BMI, SBS etiology, presence of a stoma, small bowel length, PS dependency, and isolated small bowel (ISB) transplant status. Dependency on PS was measured through combined parenteral nutrition (PN) and IV fluid volume (mL/kg/wk), days per week, hours per day, caloric intake (cal/kg/d), parenteral nutrition dependency index (PNDI), and lipid dose (g/kg/d).

PNDI is the ratio between non-protein energy intake (NPEI), or energy provided by parenteral nutrition, and resting energy expenditure (REE), estimated using the Schofield equation. PNDI serves as an indirect measure of intestinal absorption. PS dependency can be classified as “mild” (PNDI <80 %), “high” (80–120 %), or “very high” (>120 %). [4]

PS dependency was recorded at initiation of teduglutide therapy and at most recent clinical visit or at time of teduglutide discontinuation, if applicable. The primary end point was a reduction in PS volume of 20–100 %. Secondary end points included changes in days receiving PS, hours receiving PS, PNDI, PS nonprotein calories, and lipid dose. Subgroup analysis comparing patients below and above age 5 at baseline was also performed.

### Statistical analysis

Descriptive statistics were used to characterize patients. Continuous variables were reported as mean with standard deviation (SD).

## Results

### Patient characteristics

We identified 32 patients of both sexes ages 1–23 years who received teduglutide for treatment of SBS between 8/2019 and 12/2024. Exclusion of patients due to noncompliance (n = 2), lost to follow-up (n = 2), and no documented PS dependency at baseline (n = 1) was conducted. Adverse events were reported on all patients and classified as “serious” if they resulted in hospitalization. The relatedness of adverse events was determined through clinician input found in medical records.

A final cohort of 27 patients were identified. Baseline characteristics were collected and summarized in Table 1. Mean age at baseline was 8.90 (SD=5.22) years. The most common SBS etiologies included gastroschisis (37 %), necrotizing enterocolitis (26 %), volvulus (11 %), and Hirschsprung’s Disease (11 %). A stoma was present for 15 % of patients. Mean small bowel length was 56.9 (SD=52.1) cm. Mean continuous period of PS dependence prior to teduglutide initiation was 3.70 (SD=3.10) years. 5 were listed for ISB transplant, 2 were “inactive,” and 20 were not listed. 2 patients were post-enterectomy. Patients received teduglutide for mean 120.8 (SD=80.7) weeks.

**Table 1:** Demographic characteristics at baseline.

Age, mean (SD)	8.90 (5.22)
Age range	1.35 – 23.13
BMI, mean (SD)	16.5 (1.96)
Cause, n (%)	
Gastroschisis	10 (37 %)
Necrotizing Enterocolitis	7 (26 %)
Hirschsprung’s Disease	3 (11 %)
Volvulus	3 (11 %)
Other	4 (15 %)
Female, n (%)	10 (37 %)
Patients with stoma, n (%)	4 (15 %)
Types of stoma, n (%)	
* Jejunostomy*	*2 (50 %)*
* Ileostomy*	*1 (25 %)*
* Colostomy*	*1 (25 %)*
Colon in continuity, n (%)	23 (85 %)
Remnant small bowel length, mean (SD), cm	56.9 (52.1)
PS total daily volume, mean (SD), mL/kg/wk	449.67 (220.13)
Median	394.37
Range	83.13–867.45
Days receiving PS, mean (SD), d/wk	6.93 (0.38)
Median	7
Range	5–7
Hours receiving PS, mean (SD), hrs/d	11.67 (3.10)
Median	12.00
Range	6–18
PS nonprotein calories, mean (SD), cal/kg/d	28.94 (15.36)
Median	25
Range	6–61
PNDI, mean (SD), %	62.63 (28.33)
Median	55.00
Range	13–116
Lipid volume, mean (SD), g/kg/d	1.09 (0.89)
Median	1.00
Range	0–2.76

### Efficacy

#### Primary end point

The primary end point was a reduction in PS volume of 20–100 %. 18 patients (67 %) achieved this end point, with 8 patients (30 %) successfully weaning from PS completely.

#### Secondary end points

Secondary end points included a significant reduction in PS volume (-150.82 mL/kg/wk, p = 0.0087), hours/day receiving PS (-3.37 h, p = 0.0014), days/week receiving PS (-2.22 days, p = 0.0013), nonprotein calories received through PS (-8.78 cal/kg/d, p = 0.0022), PNDI (-19.67 %, p = 0.0016), and lipid dose (-0.34 g/kg/d, p = 0.0495). Indicators of treatment response at baseline and final follow up are detailed in Table 2.

**Table 2:** Indicators of treatment response.

	Baseline (SD)	Last follow up (SD)	Difference (p-value)
**PS volume (mL/kg/wk)**	449.67 (220.13)	298.85 (309.77)	-150.82 (0.0087)
**PS days/week**	6.93 (0.38)	4.70 (3.11)	-2.22 (0.0013)
**PS hours/d**	11.67 (3.10)	8.39 (6.21)	-3.37 (0.0014)
**PNDI (%)**	62.63 (28.33)	43.0 (38.0)	-20.0 (0.0016)
**PS nonprotein calories (cal/kg/d)**	28.94 (15.35)	20.16 (19.45)	-8.78 (0.0022)
**PS lipid dosage (g/kg/d)**	1.09 (0.89)	0.75 (0.86)	-0.34 (0.0495)
**BMI**	16.5 (1.96)	17.01 (2.78)	0.55 (0.1470)

Of the 5 patients listed for ISB transplant at baseline, 2 remained listed, 2 were removed, and 1 was reclassified as “inactive.” 1 was newly listed and 2 “inactive” patients were removed. 19 patients remained unlisted. The 2 patients who received teduglutide post-enterectomy experienced significant decreases in PS volume (46.5 % and 43.7 %).

### Successful PS weaning

**Fig. 1:**
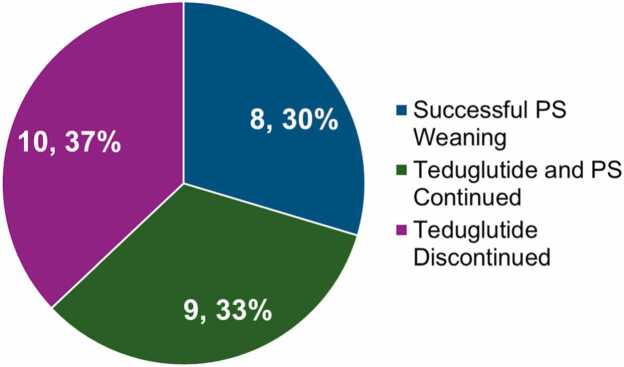
Distribution of patient status at final follow-up: successful PS weaning, continued teduglutide treatment and PS, or discontinued teduglutide treatment.

**Fig. 2:**
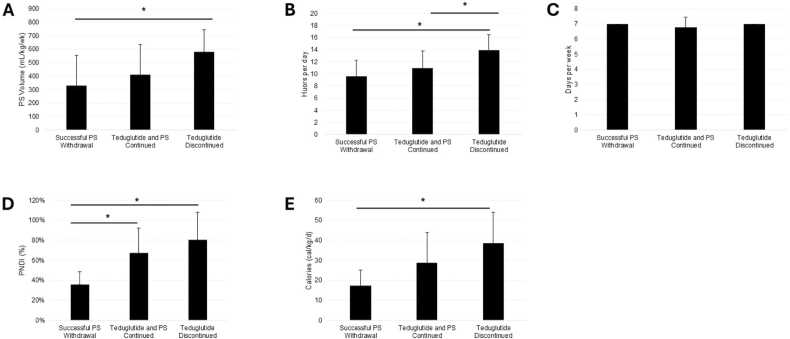
Baseline characteristics of patients categorized by outcome of teduglutide treatment. (A) Weekly PS volume. (B) Hours per day receiving PS. (C) Days per week receiving PS. (D) PNDI. (E) PS nonprotein calories.

8 patients (30 %) successfully weaned to intravenous fluids at mean 45.35 (SD=59.28) weeks and achieved enteral autonomy at mean 71.57 (SD=56.37) weeks. At teduglutide initiation, these patients had a significantly lower PS volume (p = 0.0150), PS hours per day (p = 0.0032), PNDI (p = 0.0006), PS nonprotein calories (p = 0.0027), and PS lipid dose (p = 0.0017) at baseline than patients who ultimately discontinued teduglutide treatment (Fig. 1, Fig. 2). Characteristics of these patients are described in Table 3.

**Table 3:** Characteristics of patients who completed successful PS withdrawal.

Patient	Sex	Age at baseline, years	Years of TPN Dependency prior to Teduglutide start	SBS Etiology	Small Bowel Length, cm	Stoma Present	Baseline PN Total Volume, mL/kg/d	Baseline PN hrs/day	Baseline PN days/wk	Baseline PN Nonprotein Calories, cal/kg/d	Baseline PNDI (%)	Weeks to Wean to IVF	Weeks to Attain Enteral Autonomy
1	F	8.15		Volvulus	13	No	174.8168	7	7	6	15 %	NA	35.86
2	M	5.11	1.06	Necrotizing Enterocolitis	110	No	394.3662	14	7	24	48 %	24	70
3	M	18.7	0.27	Kabuki syndrome	45	No	116.0622	8	7	9	31 %	30.14	90.14
4	M	6.98	2.12	Gastroschisis	85	No	781.9149	11	7	18.8	29 %	24	61
5	M	5	0.88	Gastroschisis	Unknown	No	327.6224	10	7	27	44 %	178.86	199.43
6	F	12.34	0.2	Necrotizing Enterocolitis	15	No	83.12803	6	7	9	25 %	9.29	14.29
7	M	8.86	0.84	Gastroschisis	75	No	315.4065	12	7	23	53 %	21.43	47.43
8	F	4.56	1.22	Gastroschisis	15	No	449.4828	9	7	20.5	39 %	29.71	54.43

BMI did not change significantly between baseline, PS withdrawal, and most recent clinic visit at mean 181.95 (SD=63.31) weeks.

### Teduglutide and PS continued

9 patients (33 %) continued teduglutide treatment without complete PS withdrawal at mean 131.30 (SD=83.07) weeks after teduglutide initiation. These patients experienced a significant reduction in the number of hours per day receiving PS (-2 h, p = 0.0039) and in lipid dose (-0.69 g/kg/d, p = 0.0345). No patients discontinued lipids. 5 patients (56 %) achieved a significant reduction in PS volume of ≥ 20 %. BMI did not change significantly from baseline (0.72, p = 0.1925). Nonsignificant decreases in PS volume, days per week, nonprotein calories, and PNDI were observed.

### Teduglutide discontinued

Teduglutide treatment was discontinued for 10 patients (37 %) at mean 62.59 (SD=55.55) weeks. PS volume, hours per day, PNDI, nonprotein calories, and lipid dose were significantly higher for patients who discontinued teduglutide compared to those who successfully weaned from PS. 4 patients discontinued teduglutide for reasons related to treatment (2 stomal prolapse, 1 increased ostomy output, 1 gallstones), 6 for unrelated reasons (2 unrelated infections and hospitalizations, 4 electively or due to plateaus in improvement). 5 patients (50 %) achieved a significant reduction in PS volume of ≥ 20 %. BMI did not change significantly over the course of teduglutide treatment.

### Subgroup analysis by age at baseline

Subgroup analysis was performed comparing patients below and above the age of 5 at baseline. 8 patients (35 %) were under age 5 at the time of teduglutide initiation. These patients experienced a nonsignificant decrease in PS volume (p = 0.6172) whereas older patients experienced a significand decrease (p = 0.00162). Older patients also experienced significant decreases in days receiving PS, PNDI, and nonprotein calories provided by PS.

### Safety

Adverse events were reviewed for 32 patients, including patients excluded from final analysis. 29 patients (90.6 %) experienced a total of 112 AEs over the course of treatment. AEs recorded for > 5 % of teduglutide-treated patients are listed in Table 4. Most frequently noted AEs include pyrexia (excluding central line infection) (50 %); central line systemic infection (47 %); abdominal pain, (19 %); vomiting (16 %), diarrhea (13 %), stomal prolapse (9 %). 94 events (85 %) were considered serious. 11 events (10 %) were related to teduglutide treatment (Table 5).

**Table 4:** Adverse events recorded for > 5 % of Teduglutide-treated patients.

Adverse event	Patients (n = 32) (%)
All AEs	29 (91)
Pyrexia (excluding central line infection)	16 (50)
Central line systemic infection	15 (47)
Abdominal pain	6 (19)
Vomiting	5 (16)
Diarrhea	4 (13)
Prolapse of intestinal stoma	3 (9)
Catheter complication	2 (6)
Acidosis	2 (6)

**Table 5:** Adverse events related to teduglutide treatment.

Adverse event	Occurrences (n = 10)
Abdominal Pain	3
Prolapse of intestinal stoma	2
Vomiting	1
Nausea	1
Colonic obstruction	1
Peripheral swelling	1
Inflammation, joint pain	1
Gallstones	1

### Narratives of patients discontinuing teduglutide because of an adverse event considered related to treatment

**Patient 1.** This was a one-year-old patient with Hirschsprung’s Disease and a jejunostomy. 20 weeks after teduglutide initiation, the patient experienced an approximately 3-inch stomal prolapse requiring repeat reduction and subsequent hospitalization for monitoring. Teduglutide was stopped due to known associations with intestinal and stomal obstructions.

**Patient 2**. This was a nine-year-old patient with Hirschsprung’s Disease and a jejunostomy. 19 weeks after teduglutide initiation, the patient experienced significant worsening of his stomal prolapse. Teduglutide was stopped; however, the stomal prolapse required surgical correction.

**Patient 3**. This was a three-year-old patient with Hirschsprung’s Disease and an ileostomy. 7 weeks after teduglutide initiation, the patient was admitted with increased ostomy output. Biopsies revealed villous blunting, crypt hyperplasia, and an increase in inflammatory cells in the lamina propria. Teduglutide was discontinued after increased ostomy output was assessed to be secretory diarrhea.

**Patient 4**. This was an 11-year-old patient with volvulus who received teduglutide for 72 weeks. The patient developed obstructive gallstones requiring endoscopic retrograde cholangiopancreatography. Teduglutide was discontinued as there is a reported association with gallstones.

## Discussion

In this retrospective review, teduglutide was effective in reducing dependency on parenteral support. 67 % of patients achieved the primary end point defined as a reduction in PS volume of ≥ 20 % from baseline and 30 % achieved enteral autonomy. This finding is consistent with randomized controlled trials in pediatric populations. [3], [5], [6] Treatment outcome (reaching enteral autonomy versus discontinuing treatment) was associated with the level of dependency on PS at baseline. Patients who achieved enteral autonomy had significantly lower PS volume, hours per day, PNDI, nonprotein calories, and lipids compared to those who discontinued teduglutide. Successful PS weaning was not observed in patients with a PNDI value over 53 %, indicating that a higher PNDI may predict unsuccessful weaning. Cumulatively, these results suggest that reaching a certain degree of freedom from PS without teduglutide may serve as a predictor of complete discontinuation with teduglutide. This may be more strongly indicated by minimal caloric needs compared to fluid needs as the difference in caloric needs for those that successfully achieved enteral autonomy seemed more notable. Baseline PNDI values for the 8 patients who achieved enteral autonomy ranged from 15 % to 53 %. A control group was unable to be established due to the center’s practice of enrolling all eligible patients onto teduglutide. In this case, a control group would have consisted of patients with major complications in which teduglutide was contraindicated.

Subgroup analysis by age indicated that older patients experienced more significant decreases in PS dependency compared to patients under age 5.

Lipid dosage decreased significantly over the course of treatment. None of the patients who continued PS were able to discontinue lipids, suggesting that lipid discontinuation may indicate a patient is nearing enteral autonomy. Perhaps normal lipid metabolism to avoid biochemical essential fatty acid deficiency is a precursor to successful weaning from parenteral support that requires further investigation. Furthermore, lipids delivered through parenteral nutrition solution may contribute to liver injury, subsequently necessitating transplantation. [7] While we hypothesized that in some instances teduglutide may decrease liver damage and thus eliminate liver transplant need, there have been no such examples in clinical practice in our institution.

Patients who discontinued teduglutide treatment experienced modest decreases in PS volume, PNDI, and PS nonprotein calories over the course of treatment. 50 % of these patients achieved the primary end point, comparable to the rate observed in patients who continued to receive teduglutide treatment and PS at the most recent clinic visit. These findings suggest that teduglutide may have had some clinical value for these patients by contributing to PS weaning. One challenge in determining appropriate teduglutide use has been valuing these more modest reductions in PS against the high cost of the drug itself. While population-based models suggest that teduglutide use may not reach traditional cost-effectiveness thresholds, individual considerations focused on the value of more modest changes in parenteral support may alter those considerations, especially for children in whom a modest reduction in parenteral support may facilitate school attendance, developmentally appropriate activities, or other significant contributors to quality of life. [8], [9], [10]

Teduglutide was generally well-tolerated, with 4 patients (15 %) discontinuing treatment due to related adverse events. The most frequently recorded AEs included pyrexia and central line systemic infections, which are to be expected for this population due to the nature of SBS and the age of patients. Most events of pyrexia resulted in hospitalization due to patients’ vulnerability to life-threatening infection. These events were consequently classified as SAEs; however, none of these events were considered related to teduglutide treatment. Pyrexia, central line infections, and abdominal pain occurred at similar rates in previously published studies in pediatric populations. [11] Vomiting was observed less frequently in this report than in many publications. This could be due to the retrospective nature of this report and limited frequency of clinic visits in some cases. The two cases of stomal prolapse, including one where surgical correction was required, do lead to some pause in our consideration for teduglutide use in children with severe type 1 anatomy with limited likelihood of achieving meaningful parenteral support reduction. Additionally, 3 patients with Hirschsprung’s disease were included in the study but all discontinued teduglutide use at mean 15.9 weeks. This was likely due to the presence of a small bowel stoma in two of these patients and subsequent stomal prolapse.

While limitations of this case series include its retrospective nature at a single center, the relatively large sample size adds to the growing body of evidence supporting GLP-2 analogues. In particular, we propose two potential indicators of successful teduglutide therapy when attempting to predict enteral autonomy achievement: baseline PNDI and lipid discontinuation while receiving teduglutide. Future prospective work may utilize these along with other suggested indicators in the literature, such as baseline oral intake, to develop more realistic decision support for children considering teduglutide therapy. [12]

## Conclusion

In conclusion, this real-world assessment of pediatric patients with SBS treated with teduglutide demonstrated significant reductions in PS dependency over mean 120.8 weeks, with 30 % achieving enteral autonomy.

## Ethical clearance

The study was approved by the University of Pittsburgh Institutional Review Board (STUDY25020106).

## Patient's/ Guardian's consent

Not applicable.

## CRediT authorship contribution statement

**Claire Josey:** Writing – review & editing, Writing – original draft, Investigation, Data curation, Conceptualization. **George Mazariegos:** Writing – review & editing, Supervision, Conceptualization. **Elizabeth King:** Data curation. **Pamela Holzer:** Data curation. **Jeffrey Rudolph:** Supervision. **Vikram Kalathur Raghu:** Writing – review & editing, Writing – original draft, Supervision.

## Funding

This research did not receive any specific grant from funding agencies in the public, commercial or not-for-profit sectors. Dr. Raghu was supported by the National Center for Advancing Translational Sciences of the National Institutes of Health under Award Number KL2TR001856. The content is solely the responsibility of the authors and does not necessarily represent the official views of the National Institutes of Health.

## Declaration of Competing Interest

Dr. Raghu serves on a data safety committee for Cormedix.
